# Development of the set of resource use items to capture costs related to informal care for people with Rare Dementia for RD‐TALK

**DOI:** 10.1002/alz.092682

**Published:** 2025-01-09

**Authors:** Katherine Cullen, Emilie V Brotherhood, Deborah Fitzsimmons, Joshua Stott

**Affiliations:** ^1^ Swansea University, Swansea, Wales United Kingdom; ^2^ Dementia Research Centre, UCL Queen Square Institute of Neurology, University College London, London United Kingdom; ^3^ Department of Clinical, Educational, and Health Psychology, Division of Psychology and Language Sciences, University College London, London United Kingdom

## Abstract

**Background:**

Non‐memory‐led dementias pose additional challenges to ‘typical dementias’ including unusual symptoms and younger onset leading to particularly high neuropsychiatric comorbidities. As part of the economic evaluation supporting the RD‐talk research programme, we require the collection of participant level resource use associated with the intervention compared with usual care. The resource use measure (RUM) needs to be sufficiently comprehensive, but still focused to capture the key items of interest (e.g., main drivers of resource use and associated cost) to balance the trade‐off between precision, effort, and burden to research participant.

**Method:**

Initial discussion with carers of people with non‐memory‐led dementia and clinical experts informed a draft list of resource items to design a RUM. A modified Delphi survey was carried out with informal carers and healthcare professionals (HCPs). Consensus of each item, using pre‐defined levels to keep or remove items, was summarised using descriptive statistics and percentage agreement. Inter‐rater reliability between rounds was assessed using the intra‐class correlation coefficient; the stability of the raters’ responses was assessed with the Wilcoxon signed rank test.

**Result:**

For round one, 18 participants were recruited (11 HCPs, 9 carers), 14 participants remained for round two (7 HCPs, 7 carers). For primary/community care, general practitioner appointments, psychological interventions (such as talking therapy), and rare dementia support groups or direct calls were all highly rated by both HCPs and carers. The RUM should include time taken off work or taking early retirement due to caring for someone with rare dementia. Categories of caring tasks were listed, from personal care and physical help, to helping with paperwork, financial matters, dealing with care services and benefits, and managing challenging behaviour. All categories were highly rated by HCPs and carers.

**Conclusion:**

Resource use measures are mainly designed for the person with a disease or condition and focus on health and social care use. There can be a considerable burden on informal carers when someone is diagnosed with rare dementia, and it is important to capture key resource items in addition to health and social care relevant to this population when evaluating interventions in rare dementia management.

